# Suicidal Behavior and Medication Adherence in Schizophrenic Patients

**DOI:** 10.7759/cureus.12473

**Published:** 2021-01-04

**Authors:** Zain I Warriach, Marcos A Sanchez-Gonzalez, Gerardo F Ferrer

**Affiliations:** 1 Department of Psychiatry, Larkin Community Hospital, Miami, USA

**Keywords:** schizophrenia, suicide, medication adherence strategies

## Abstract

Suicide is one of the potential complications in the Schizophrenic patient population. This review article deals with the significance of antipsychotic medication compliance in decreasing suicidal behavior and hospitalizations in Schizophrenic patients. The medication adherence with second-generation antipsychotics (SGA) like clozapine and long-acting injectables (LAIs) like paliperidone is associated with decrease suicidal behavior and all-cause mortality in Schizophrenic patients. Concomitant treatment of depression and substance abuse disorder in this patient population is also associated with decreasing all-cause mortality and hospitalizations. On the other hand, long-term benzodiazepine use is associated with increase mortality in Schizophrenic patients. We also discuss some important physician intervention strategies to improve medication adherence in Schizophrenic patients like motivational interviewing (MI), behavioral tailoring (BT), and psychosocial interventions like cognitive behavior therapy (CBT).

## Introduction and background

Schizophrenia is a neuropsychiatric disorder affecting millions of people worldwide. It is a very serious debilitating mental health disorder affecting the thoughts, perceptions, feelings, and behavior and making the patient disconnect from reality. Suicide is one of the serious and lethal outcomes in the Schizophrenic patient population [1]. Around 10% of the Schizophrenic patient population dies due to suicide. There are several risk factors accountable for the suicidal behavior in this patient population [2-6]. The most common ones are white ethnicity, younger age, unmarried, higher intellectual level, social isolation, gradual onset of illness, hopelessness, co-morbid substance abuse, previous hospitalizations, previous suicide attempts, family history of suicide, family stress, impulsivity, positive symptoms of schizophrenia, unemployment, prolonged illness, the greater insight of the illness and others [7-11]. We conducted this review article to determine the significance of antipsychotic medication compliance in Schizophrenic patients to reduce suicidal ideation and hospitalizations. Second-generation antipsychotics (SGA) like clozapine and long-acting injectable antipsychotics like paliperidone have reached statistically significant value and proven to decrease suicidal behavior in the Schizophrenic patient population [12-17]. Concomitant treatment of depressive symptoms in the Schizophrenic patient population with citalopram is also associated with decreasing all-cause mortality and hospitalizations [18-24]. Further, we discuss the patient factors causing non-compliance like medication side effects, hopelessness, lack of social support and motivation, social stigma, skepticism of benefits, and others. We further elaborate on the physician's role in improving medication compliance by adopting motivational interviewing (MI) techniques, behavioral tailoring (BT) strategies, and psychosocial interventions like cognitive behavior therapy (CBT).

## Review

Methods

This is a traditional review, not a systematic review, so no Preferred Reported Items for Systematic Reviews and Metal-Analysis (PRISMA) or Measurement Tool to Assess Systematic Reviews (AMSTAR) guidelines are used. The electronic search for reference databases was performed by using PubMed as the search engine. No inclusion and exclusion criteria are used for the review article. Regular key-words- “Schizophrenia” AND “Suicide” AND “Antipsychotics” yield 5498 articles on PubMed. The Medical Subject Headings (MeSH) keywords for these searches yield no articles. As healthcare professionals, we collected and used all the data ethically.

Discussion

Suicidal behavior is a common complication in Schizophrenic patients and has various risk factors associated with this problem [1-3]. The most common ones are younger age, higher intellectual level, depression, hopelessness, and greater insight into the illness. Among others most notable are previous suicide attempts, family history of suicide, white male, unemployment, impulsivity, positive symptoms of schizophrenia, and others [4-7]. The evaluation of the risk factors and predicting the suicidal outcome in the schizophrenic patient population is a tough task for the physician. However, knowing these possible risk factors helps the physician better evaluate and address patient beliefs and values related to the disorder [8-11].

**Figure 1:**
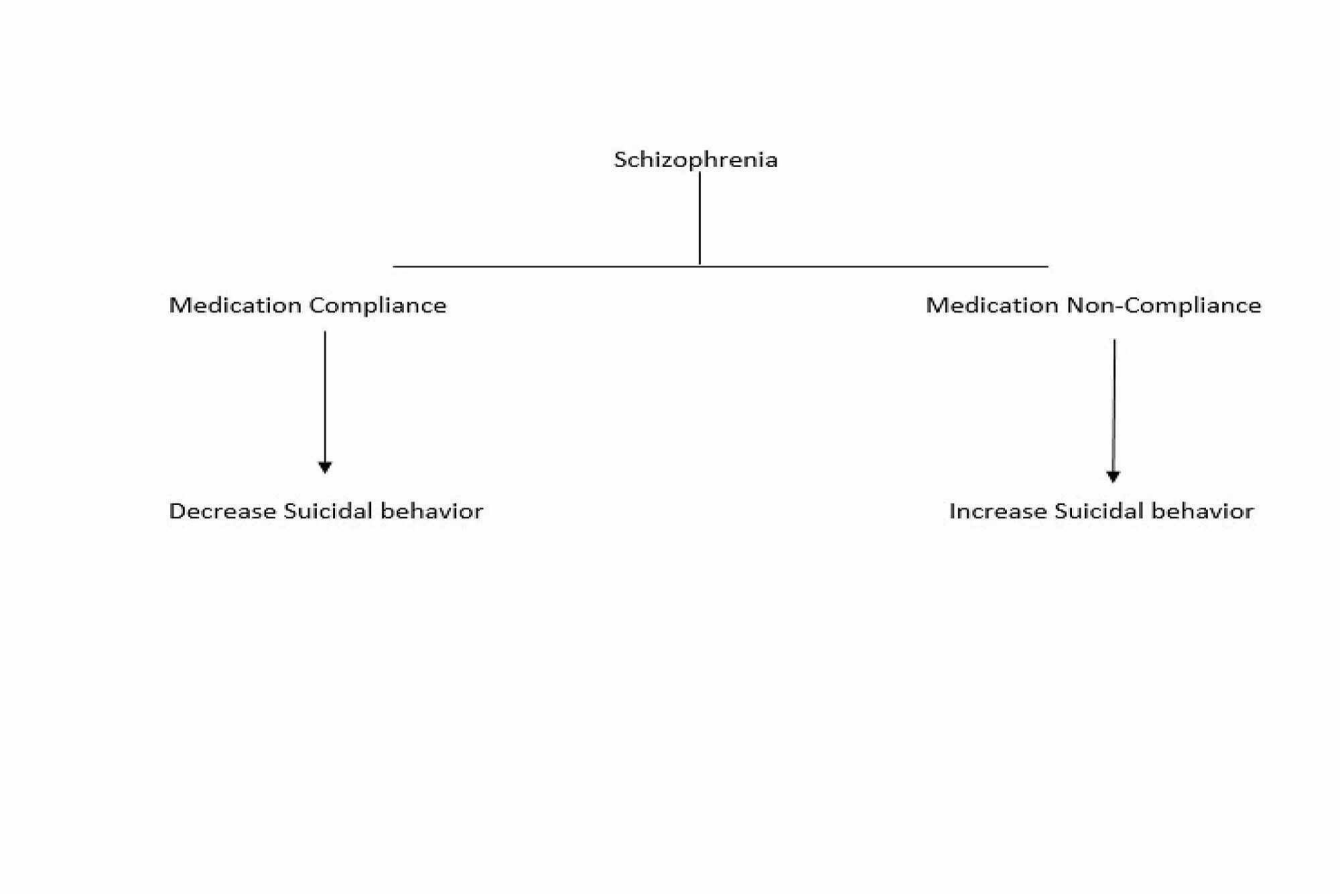
Suicidal behavior and medication compliance in Schizophrenia.

Our literature study reveals the efficacy of antipsychotic medication compliance in reducing suicidal behavior in the schizophrenic patient population. We did an extensive literature review and found that SGA like clozapine has been very effective in reducing suicidal behavior, all-cause mortality, and hospitalization. No other SGA including olanzapine has shown any superiority over clozapine in reducing suicidal behavior [12-15]. The effects of clozapine in reducing suicidal behavior and hospitalizations comes from its intrinsic pharmacology and not from concomitant psychotropic medications used. Further studies revealed that long-acting injectable (LAI) SGA like paliperidone have shown promising results in reducing hospitalizations and all-cause mortality [16-18]. Concomitant depression and substance use disorder should also be addressed thoroughly in Schizophrenic patients. Literature studies revealed that the use of antidepressants like citalopram along with antipsychotics has been effective in decreasing hospitalizations and mortality from all causes including suicidal behavior. The use of benzodiazepines has been warranted on many occasions for treating patients diagnosed with Schizophrenia. On the other hand, long-term benzodiazepine use in the schizophrenic patient population has been associated with more hospitalizations and increase mortality [19-27]. Figure 1 shows the association of medication compliance and suicidal behavior in Schizophrenia patients.

From a review of the literature, we also elaborate on some of the patient factors causing non-compliance in Schizophrenic patients. One of the perspectives from the patient side is medication side effects resulting in non-compliance [28,29]. Most of the psychiatric medications take anywhere from weeks to months to be efficacious resulting in the patient's fear of both original symptoms and side effects from the medication. Social stigma, another factor that is perceived as being an inferior stereotype for the society by psychiatric patients and creates a sense of rejection and alienation from the society, leading towards more self-stigma and internalized stigma of mental illness. This self-stigma and fear of rejection start taking intangible control of their mind resulting in poor medication compliance. Some Schizophrenic patients are also skeptical of the benefits of these long-term medications. Forgetfulness, cost, and dependency on psychotropic medications are some other patient factors for medication non-compliance. Hopelessness, lack of social support and motivation in this patient population also contribute to medication non-compliance. Table 1 highlights the important studies relevant to the review article.

**Table 1:** Important studies relevant to the review article.

Studies	Schizophrenia	Antipsychotics compliance	No compliance	Suicidality/mortality outcome
20-year follow-up study of physical morbidity and mortality in relationship to antipsychotic treatment in a nationwide cohort of 62,250 patients with Schizophrenia (FIN20) (Taipale et al. 2020 [12])	Yes	Yes	N/A	The data suggest that patient treated with clozapine is associated with lower all-cause mortality and hospitalizations.
Polypharmacy with antipsychotics, antidepressants, or benzodiazepines and mortality in Schizophrenia (Tiihonen et al. 2012 [19])	Yes	Yes	N/A	Benzodiazepine use was associated with increase mortality while antidepressants and antipsychotics were not associated with increase mortality.
Patterns of concomitant psychotropic medication use during a two-year study comparing clozapine and olanzapine for the prevention of suicidal behavior (Glick et al. 2004 [23])	Yes	Yes	N/A	The effects of clozapine in reducing the risk of suicidal behavior derive from its intrinsic pharmacology and not from the influence of concomitant psychotropic medications.
Real-world effectiveness of antipsychotic treatments in a nationwide cohort of 29,823 patients with schizophrenia (Tiihonen et al. 2017 [14])	Yes	Yes	N/A	The risk of rehospitalization is about 20% to 30% lower during long-acting injectable treatments compared with equivalent oral formulations.

In this literature review, we searched some studies putting a strong emphasis on certain physician interventions leading to improved medication adherence. Motivational interviewing (MI) is one of the most important physician factors in improving medication adherence [30-33]. A trusting relationship and empathy enable both the physician and the patient to attain depth in the conversation and this fruitful interaction gives opportunities to trigger mechanisms of change of behavior in the patient toward adherence. When the patient is not equivocal, this MI supports the exploration of compliance behavior in relation to the patient’s beliefs and goals, strengthening long-term motivation in the patient and exploring patient perspectives on the benefits of long-term medication use [34-36]. Among the Schizophrenic patient population, where the disease management factors are difficult to change, the motivation for medication use is an important tool for medication compliance [37]. For this reason, it is very important for physicians to practice empathy in their patient interactions. They should explore the patient’s attitude towards medications, whether they have any perceived notions, fears, or hope regarding a particular drug. Drug Attitude Inventory-30 (DAI-30) scoring is an important tool to indicate positive or negative attitudes toward psychiatric medications and serves as a basis to guide discussions with patients [38]. Shared decision making by directly involving the patient in making their own decisions shows a positive therapeutic alliance and medication compliance in patients. The patient should be encouraged to search for the recommended medications or any alternatives on their own and then participate in a discussion of the pros and cons. If the patient is experiencing any side effects from a particular drug, then the patient should be switched to another medication. The physician should also help in simplifying the medication regimen if it is too difficult for the patient to understand. A psychosocial intervention like CBT is a recommended treatment modality nowadays besides medication for Schizophrenia [39,40]. It helps to evaluate the distressing and problematic behavior of the patients thinking, their self internalized stigma regarding diagnosis, anxiety, and fear of dependency on the medication. Addressing all of these concerns with open communication between the clinician and the patient could lead to better compliance. The physician should address the patient fear of dependency on long-term medications. When patients are devoid of symptoms and feeling fine, they start believing they have made a full recovery from the disorder and are not in need of further medication. The physician should tell them the importance of long-term medication compliance to prevent relapse of symptoms. 

BT is another group of cognitive-behavioral strategies in patients with serious mental illnesses like Schizophrenia to improve medication compliance and overall wellbeing [41]. BT is a multistep process that begins with an open conversation between the clinician and the patient to identify the goals, needs, and supports of the patient. Exploring the patient’s daily routine and activities including meals, hygienic practices, and identifying the various ways medications can be integrated into their lifestyle, can help prompt a change in their behavior. After developing the BT strategy, the physician should demonstrate it to the patient during sessions and encourage the individual to participate in it as well and practice between sessions and monitor for any change in behavior in the following sessions. Some non-technology-based BT strategies that can promote medication adherence include environmental supports at home, pharmacy-based reminders by mail, telephonic reminders by health care providers, and some modest financial incentives. We also explore new technology-based BT strategies for improving medication adherence. Smartphone applications can help individuals in setting their medication and dosage schedule and place reminders for the next dose [42,43]. Patients with access to portal software and websites can request refills and may better adhere to their medication regimen. The electronic pillbox is a new innovative technology that tracks patient medication intake. If there is any missed dose, the patient and the family member will receive a text message alert. Electronic pill bottle caps that have embedded software are able to detect every time the cap is opened or closed. In addition, when it's time for refills, users can view the compliance data on a mobile device [44]. Although all these technologies and non-technology-related strategies help mentally ill patients in improving their medication adherence, there are still limitations to them including cost, data recovery, recruitment of more mental health providers, and their practical implementation. However, several difficulties are encountered in this patient population, more ways and strategies should be developed to improve adherence by improving data recovery, frequent follow-ups with the healthcare provider, good coordination between physicians and pharmacies, and improving cost-effectiveness in implementing the use of these BT strategies. This review would be able to assist physicians to choose the most suitable behavioral strategy specifically aimed towards the patient’s particular goals and improving medication adherence behavior.

## Conclusions

This literature review emphasizes the importance of using SGA like clozapine and SG-LAI antipsychotics in decreasing suicidal behavior, rehospitalizations, and all-cause mortality in a group of Schizophrenic patient populations. Concomitant treatment of depression and substance use disorder has also proven effective in decreasing hospitalizations and all-cause mortality. Medication compliance is a big challenge in mentally challenged patients. We further discuss some of the physician interventions like MI, BT, and psychosocial interventions like CBT which could be helpful in improving medication adherence behavior in Schizophrenic patients. 
